# Genomics and infectious disease: a call to identify the ethical, legal and social implications for public health and clinical practice

**DOI:** 10.1186/s13073-014-0106-2

**Published:** 2014-11-18

**Authors:** Gail Geller, Rachel Dvoskin, Chloe L Thio, Priya Duggal, Michelle H Lewis, Theodore C Bailey, Andrea Sutherland, Daniel A Salmon, Jeffrey P Kahn

**Affiliations:** Berman Institute of Bioethics, Johns Hopkins University, Baltimore, MD 21205 USA; Department of Medicine, Johns Hopkins University School of Medicine, Baltimore, MD 21205 USA; Department of Health, Behavior & Society, Johns Hopkins University Bloomberg School of Public Health, Baltimore, MD 21205 USA; Department of Health Policy and Management, Johns Hopkins University Bloomberg School of Public Health, Baltimore, MD 21205 USA; Department of Epidemiology, Johns Hopkins University Bloomberg School of Public Health, Baltimore, MD 21205 USA; Department of International Health, Johns Hopkins University Bloomberg School of Public Health, Baltimore, MD 21205 USA

## Abstract

Advances in genomics are contributing to the development of more effective, personalized approaches to the prevention and treatment of infectious diseases. Genetic sequencing technologies are furthering our understanding of how human and pathogen genomic factors - and their interactions - contribute to individual differences in immunologic responses to vaccines, infections and drug therapies. Such understanding will influence future policies and procedures for infectious disease management. With the potential for tailored interventions for particular individuals, populations or subpopulations, ethical, legal and social implications (ELSIs) may arise for public health and clinical practice. Potential considerations include balancing health-related benefits and harms between individuals and the larger community, minimizing threats to individual privacy and autonomy, and ensuring just distribution of scarce resources. In this Opinion, we consider the potential application of pathogen and host genomic information to particular viral infections that have large-scale public health consequences but differ in ELSI-relevant characteristics such as ease of transmission, chronicity, severity, preventability and treatability. We argue for the importance of anticipating these ELSI issues in advance of new scientific discoveries, and call for the development of strategies for identifying and exploring ethical questions that should be considered as clinical, public health and policy decisions are made.

## Introduction

Genomic information offers the opportunity for more personalized treatment and prevention [1] in clinical practice and public health settings. Until recently, such efforts have focused largely on common, complex diseases (for example, cancers, heart disease, neurodegenerative diseases) and less common inherited diseases; examples of such efforts include risk screening, diagnostic sequencing and pharmacogenomics. Now there is growing interest in the application of genomics to the management of infectious diseases and epidemics [2], which are among the top global public health burdens [3]. Rapid and large-scale sequencing of pathogen genomes, which provides stronger and more accurate evidence than was previously possible for source and contact tracing, is being applied widely for disease outbreak management [4] - most recently and publicly in the case of the Ebola outbreak in West Africa [5,6]. Additional uses include precise diagnosis of microbial infection, describing transmission patterns, understanding the genomics of emerging drug resistance and identifying targets for new therapeutics and vaccines. There is growing evidence that, as well as pathogen genetic factors, host genetic factors and the interaction between host, vector and pathogen influence variability in infection rates, immune responses [7,8], susceptibility to infection, disease progression and severity, and response to preventive or therapeutic interventions [9,10]. As such, genomic research is improving our understanding of infectious disease pathogenesis and immune response and may help guide future vaccine development and treatment strategies [11–18].

While the past few years have seen substantial federal and private research funding for infectious disease genomics research, there has been little discussion of the possible ELSIs - for individuals, groups or larger society - of using genomic information in the management of infectious disease. This gap may be explained in part by the current paucity of scientific advances in genomics that have practical applications to infectious disease management. Although it may be premature, we must nevertheless anticipate the possibility of ELSI-associated challenges in the future. This Opinion aims to anticipate what some of these issues might be and under what conditions they could arise. We argue that these considerations - even as the science is still developing - should become part of the agenda of researchers, clinicians, policymakers and public health officials so that the benefits of genomic applications to infectious disease are maximized while potential harms to individuals and populations are minimized.

We begin by acknowledging the existing scholarship on ELSI issues in the genomics of non-communicable diseases, and the ethical and legal issues surrounding infectious disease management. Then we briefly describe some of the epidemiologic characteristics and recent genomic advances associated with four particular infectious diseases - Ebola, pandemic influenza, hepatitis B and tuberculosis - that have large-scale public health consequences but differ in terms of ease of transmission, chronicity, severity, preventability and treatability, factors which affect a range of ELSI issues. In this section we also consider the situations under which the use of genomic information might or might not be appropriate in the management of infectious diseases. Finally, we describe some of the major ethical, legal and social issues that arise in the context of genomics and how they may play out in the management of these four specific infectious diseases.

## Relevant ethics scholarship: what we know and what might be ahead?

More than two decades of ELSI research on the application of genomics to complex diseases has produced many insights that are also relevant to infectious diseases [19]. With regard to genetic susceptibility testing in a clinical setting, issues include the reliability, validity, confidentiality and disclosure of genetic information. In the case of clinical next-generation sequencing, and in genetic cohort studies and biobanks, pertinent issues include the interpretation of data, data storage, data sharing, informed consent and identifiability/privacy [20–26].

However, a number of factors are unique to infectious disease, highlighting the importance of investigating whether novel ELSI issues or variations on existing issues might emerge from the application of genomics in this context. Importantly, the nature of disease transmission differs from that in other types of disease, which has implications for who is at increased risk. Inherited forms of non-infectious diseases exhibit vertical transmission - from one generation to the next. By contrast, infectious diseases can be transmitted horizontally (in addition to vertically) to unrelated or unknown individuals, and those at risk of exposure are often unaware of their risk. In addition, in the case of infectious diseases, potential benefits or harms of healthcare policy accrue to the entire population - as in the case of vaccination - in keeping with the goals of public health. The ethical tensions between the goals and implementation of personalized medicine and those of public health, though not new, are highlighted by the application of genomics to infectious disease management.

Existing literature on infectious disease policy, ethics, and law, outside the context of genomics, describes the potential for stigmatization of individuals or subpopulations, the challenge of balancing individual interests and protections (for example, privacy, autonomy, freedom of movement) against risks of harm to others and to public health, issues of justice, and employer or health professional obligations [27,28].

**Figure 1:**
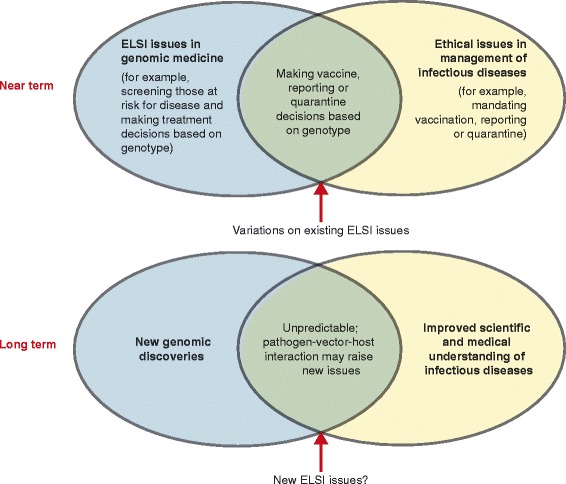
**Status of ELSI issues at the intersection of genomics and infectious diseases.** In the near term, the ELSI issues that arise at the intersection of genomics and infectious disease are likely to reflect new twists on existing ELSI challenges. In the future, as new scientific discoveries elucidate important host-vector-pathogen interactions, novel ELSI issues might emerge; implications for individuals and society are as yet unknown and unpredictable.

At the intersection of genomics and infectious diseases, there has been some discussion of the ELSIs of using pathogen genomics for source and contact tracing [29–31], but little attention has been paid to the ELSI issues regarding testing for and using host genetic information in infectious disease prevention and control. As shown in Figure 1, the introduction of genomic information to infectious disease management may complicate or exacerbate existing ELSI issues, or create variations on existing challenges for clinical practice, public health and policy making.

## Infectious diseases: epidemiology, characteristics and recent genomic advances

Infectious diseases account for a significant component of disease burden worldwide, and are responsible for a large proportion of morbidity and mortality across all areas of society [3]. Infectious diseases vary by mode of transmission (human to human, vector-borne, waterborne, and so on) and type of pathogen (for example, bacterial, viral) [2]. Infectious agents can cause acute illness (for instance, influenza) or chronic illness (such as with hepatitis B virus (HBV) and HIV), and chronic illnesses can sometimes occur with few or no symptoms until the disease has become significantly advanced.

Strategies for the clinical management and public health control of different infectious diseases vary depending on the acuteness and chronicity of infection, infectivity and virulence of the causative pathogen, modes and ease of transmission, and whether there are effective treatments, vaccines, or other means of prevention. These factors, alone or in combination, are important determinants of the ELSI issues that may arise with genomic applications to infectious disease. For example, whether a disease is transmitted among humans through casual or close contact influences who is at increased risk and whether they are aware of their risk. Or whether a highly contagious disease is preventable or treatable may influence the decision to implement liberty-limiting policies. The genomic variants associated with infectious diseases may be viewed as another characteristic that may or may not be useful in infectious disease management.

### When might genomic information be relevant or useful

When safe and effective preventive or therapeutic interventions exist, it is unwarranted - indeed, unethical - to use genomic information to stratify patients or the public for treatment or disease management; all at-risk or affected individuals should receive the intervention regardless of genotype. For example, the *CCR5Δ32* allele is associated with resistance to HIV-1 infection and delayed AIDS progression in HIV-infected individuals [32]. However, given the effectiveness of antiretroviral therapies [33], treatment would never be withheld from those who carry the *CCR5Δ32* genotype. In the case of the hepatitis C virus (HCV), *IL28B* genotype is associated with response to HCV antiviral treatment and natural clearance of the virus [34]. Until recently, the available forms of treatment were not 100% effective and were associated with burdensome injections and side effects [35]. At that time, it might have been appropriate to consider genotyping at-risk individuals and offering treatment preferentially to those least likely to clear the virus spontaneously. However, with the development of combination therapies and other highly effective treatments with few side effects [35], the individual’s genotype is now irrelevant for clinical or public health decision making. Nevertheless, there are other situations and diseases for which genomic information might be useful. We describe below the epidemiology and genomics of four particular infectious diseases - Ebola, pandemic influenza, hepatitis B and tuberculosis. We chose these diseases because of their public health significance and because, as shown in Table 1, they represent different combinations of the characteristics outlined above.

**Table 1:** **Examples of infectious diseases of varying characteristics, relevant host genomic discoveries and anticipated ELSI issues**

**Disease example**	**Characteristics**					**Host genetic association(s)**	**Illustrative ELSI issue**
	***Chronicity***	***Contact***	***Severity***	***Treatability***	***Preventability***		
Ebola	Acute	Close	Unknown; high case fatality in epidemics	No	No	None right now	Restricting civil liberties by using genomic information to inform quarantine policy or travel restrictions
							Fairness implications of genotype-based triage decisions in resource-limited settings
Pandemic influenza	Acute	Casual	Variable	Yes, but variable	Yes, but variable	Markers associated with increased susceptibility to infection, severity of disease and response to vaccine	Imposing workforce restrictions on healthcare personnel or selectively excluding students who are more likely to be super-spreaders from educational settings during a pandemic
Hepatitis B	Chronic form	Close	Often Severe	Yes, but no cure	Yes (vaccine is 95% effective)	Markers associated with vaccine non-response	Prioritizing access to therapy for vaccine non-responders based on genotype, particularly in resource-limited settings
							Exempting vaccine non-responders from job-dependent mandatory vaccination
Tuberculosis	Chronic, active form	Casual	Variable	Yes, but low efficacy, side effects and multidrug resistance	Vaccine only 20% effective	Markers associated with susceptibility to active disease in particular ethnic or geographic populations	Targeting specific, marginalized subgroups for genotyping (for example, prisoners, native populations, inner city communities) and then treating individuals differentially based on their genetic susceptibility to active infection

ELSI, ethical, legal and social implication.

#### Ebola

The recent Ebola outbreak illustrates the enormous clinical and public health challenges surrounding an infectious, high-mortality disease for which outbreaks are rare yet potentially devastating. In the past 40 years, Africa has seen a number of isolated Ebola outbreaks, but the current one, which at the time of publication had resulted in more than 4,800 deaths, is the first epidemic. Because there are few clinical or laboratory data on people infected with Ebola, we know very little about the science or epidemiology of the disease. There is currently no approved prevention or treatment other than supportive care. Because we lack serology data on people in regions of Ebola outbreaks, it is not known whether there are infected individuals who remain asymptomatic; therefore, the degree of infectivity of the virus is unknown. We do know that the risk of transmission is high in the case of direct contact with bodily fluids of symptomatic individuals (or those who have died of the illness) and that in an epidemic situation, where access to adequate health care is poor, the case fatality rate is extremely high.

Sequencing of the current strain of the Ebola virus has enabled researchers to trace the outbreak’s origin and pattern of transmission [5,6]. This technology is currently the only known genomic application to the understanding and management of Ebola virus disease. Because people exposed to Ebola show phenotypic variability in susceptibility to infection and disease severity, it is likely that human genetic variation contributes to individual immunity and infectivity and that host genetic differences are one factor among many that interact to influence the infection.

#### Hepatitis B

Hepatitis B is found in virtually every region of the globe. Of the more than 2 billion people who are or have been infected, 350 to 400 million are carriers of the chronic disease; the remainder undergo spontaneous recovery and production of protective antibodies [36]. Nearly 100% of infected infants (that is, those born to HBV-infected mothers) become chronically infected. The risk of developing a chronic infection decreases with age [37,38].

At least 30% of those with chronic HBV infection experience significant morbidity or mortality, including cirrhosis and hepatocellular carcinoma. Most people do not know they are infected until they present with symptoms of advanced liver disease, which means that infected individuals can spread the infection unknowingly, sometimes for many years. Although oral antiviral therapies are effective at stopping HBV replication, they do not cure the disease. Therefore, therapy is usually lifelong. Treatment is also complicated by the development of drug resistance and side effects. A vaccine against HBV is safe and effective in 90 to 95% of people; however, the individuals who are most at risk of becoming infected are often those with limited access to the vaccine, such as marginalized populations or people living in resource-limited countries.

There is substantial evidence that an individual's likelihood of recovering from an acute HBV infection or developing severe sequelae from infection is influenced, in part, by genes [39–45]. Candidate gene and genome-wide association studies have identified variants associated with HBV-related disease progression or hepatocellular carcinoma in various populations [46–52]. Treatment response to interferon (IFN)-α has been associated in some, but not all, studies with IFNλ_3_ polymorphisms [53]. Finally, specific gene variants (*HLA* and non-*HLA* alleles) have been associated with vaccine response and non-response [54–57].

#### Pandemic influenza

Acute viral infections such as influenza also have profound impacts on global health [58]. In contrast to the yearly epidemics caused by seasonal influenza, a pandemic can occur when a new virus emerges in a naive population and is readily transmitted from person to person [59]. The US Centers for Disease Control (CDC) estimates that the H1N1 2009 pandemic resulted in 41 to 84 million infections, 183,000 to 378,000 hospitalizations, and nearly 285,000 deaths worldwide [60]. Although the morbidity and mortality of that pandemic were lower than feared, public health professionals continuously monitor for the emergence of more virulent strains [61].

As an airborne infection, influenza is transmitted easily and quickly, and its effects can be acute, although there is wide variability in response to infection. Much of the heterogeneity in the severity of seasonal influenza infections has been attributed to the degree of acquired immunity in the population affected, patient co-morbidities and the virulence of the strain. Also, influenza epidemics and pandemics are often caused by the introduction of novel viruses for which most people have limited acquired immunity. The emergence of new strains, and the lack of cross-protection by existing vaccines, does not leave much time for vaccine development. In pandemics, including the H1N1 2009 influenza pandemic, healthy young individuals with no co-morbidities have comprised a significant proportion of fatal and severe cases [62]. These pandemics have provided an opportunity to evaluate the host innate immune response among populations without underlying background immunity.

Research has identified genetic factors associated with severity of illness due to influenza [63–65] and death from severe influenza [66]. Genetic information about immune response to influenza could inform vaccine development and distribution, and disease treatment strategies [17,67,68]. Several candidate gene studies suggest that variations in HLA class 1 and other genes contribute to differences in antibody response to influenza vaccines [15,69,70]. Ongoing experience with vaccine use has provided opportunities to learn about the potential role of genetics in vaccine safety and efficacy [71,72].

#### Tuberculosis

Tuberculosis causes 1.5 to 2 million deaths per year worldwide, second only to HIV in mortality due to an infectious disease. It is estimated that one-third of the global population has latent tuberculosis. Those infected have about a 10% lifetime risk of becoming ill with active tuberculosis; however, this risk is much higher for people whose immune system is compromised by HIV infection, malnutrition or other illness. Only the active form of tuberculosis is contagious but it is easily transmitted through casual contact. Tuberculosis occurs all over the world, but 95% of tuberculosis-related deaths occur in low- and middle-income countries [73]. The disease is only minimally preventable; the vaccine that is used in areas of high endemicity is about 20% effective [74]. Active tuberculosis is treatable (and curable), but disease control and treatment adherence are complicated by a variety of factors, including availability of healthcare resources, multidrug-resistant tuberculosis strains and potentially toxic side effects of treatment.

Gene variation has been associated with susceptibility to active tuberculosis in specific populations. For example, a particular gene variant in the promoter region of the *IL10* gene is associated with a 40 to 60% increased risk of developing active tuberculosis among Europeans and Americans [75]. Further research on host genomics is likely to identify genetic contributions to the phenotypic variability seen in tuberculosis infection, and lead to improvements in the efficacy of preventive and therapeutic interventions. Moreover, sequencing of the pathogen is being used to describe tuberculosis outbreak dynamics when traditional contact tracing cannot identify the source [76].

#### Other infectious diseases and recent genomic advances

In addition to diseases that are transmitted from human to human by air, blood or other bodily fluids, there are entire classes of globally burdensome infectious diseases that have different modes of transmission but exhibit similar variability in degrees of preventability, infectivity, transmission risk, treatability and chronicity.

Waterborne diseases, such as cholera, are a significant global public health burden and are among the most important causes of illness in areas with poor sanitation [77]. Recent genomic advances are contributing to our understanding of the emergence and spread of a multidrug-resistant cholera strain [78], for example, and helping to identify variants that might account for differences in host susceptibility to other waterborne infections such as schistosomiasis [79,80].

Vector-borne diseases, including malaria and dengue, are among the most common infectious diseases around the globe. Recent studies have identified genetic variants that account for variability in human susceptibility and severity of infection and might be useful for vaccine and treatment development in malaria [80–84] and dengue [85,86], for example.

Nosocomial infections, such as methicillin-resistant *Staphylococcus aureus* (MRSA), pose a major challenge to clinical management and health policy [87]. Recent whole-genome sequencing (WGS) of MRSA clones made it possible to trace the origin, evolution and global spread of EMRSA-15, currently the most rapidly spreading and tenacious healthcare-associated clone in Europe [88].

These are just a few examples of other types of infectious diseases for which genomic advances may play a role in prevention and control, with corresponding ELSI issues.

## ELSI challenges in genomics and infectious disease

The ELSI issues associated with at least one application of genomics to infectious disease management have received some attention. The ability to identify a human source of infection or a ‘super-spreader’ creates potential questions of blame or legal liability, stigmatization, and risks to privacy [29,30]. Similar issues could arise from the ability to identify people at a higher risk for contracting or spreading a disease using human genetic markers. Below we explore some of the key ethical and social considerations, as well as legal and policy considerations, that are relevant to host genomic discoveries, followed by particular examples of ELSI issues that may arise if we apply genomic discoveries to four specific infectious diseases that differ in a number of ELSI-relevant characteristics (Table 1).

### General ethical and social considerations

In the context of any technological advances in biomedical science, ethical challenges often arise when there is a lag time between the ability to identify a problem and the capacity to address it. In the case of infectious diseases, we may be able to identify those at increased risk of contracting or transmitting infection, or those more or less likely to respond to interventions, before we have safe and effective interventions to offer, or before policy can be modified. Another major ethical challenge results from the variability in the predictive value of genotypic information and how such information can be used to inform risk management policy when our understanding of risk is inexact. The significance of genomic information, and the uses to which it is put, may give rise to the following specific ELSI-related concerns: (1) an imbalance in health-related benefits and harms to individuals and populations; (2) privacy and confidentiality of personal information, autonomy, choice and limitations on liberty; (3) the social and behavioral impact of genomic information on individuals, family members and others; and (4) the equitable distribution of scarce resources. Although these issues are not unique to infectious diseases, they need to be considered as our scientific understanding of the role of genomics in infectious disease management advances. What may be unique at the intersection of genomics and infectious disease control are ethical challenges that stem from the inherent tension between the goals of personalized medicine, which are to benefit particular individuals, and those of public health - to benefit and protect entire populations.

### Benefits and harms to individuals and populations

The potential for risk, as well as benefit, is inherent in scientific discovery. One of the ethical justifications for incorporating biomedical advances in clinical practice and public health is that the benefits to individuals and/or populations outweigh the potential harms. Moreover, specific subgroups of the population should not disproportionately reap the benefits or shoulder the burdens of harm. Genomic discoveries related to infectious disease have the potential to benefit at-risk and affected individuals, and minimize harm to them, by identifying more effective preventive or therapeutic interventions and clarifying whether a pathogen or the treatment accounts for an adverse reaction to an intervention. An intervention would be ethically justified if the likelihood of an effective immune response significantly outweighs the risk and severity of adverse reactions to the intervention. It has been suggested that targeting therapeutic interventions to those more likely to develop severe illness and then protecting them from adverse reactions could be useful in pandemic planning [89]. In the context of prevention, genomic discoveries could also be used to minimize vaccine-associated adverse events, and augment immune responses in individuals who would otherwise have low or no response to vaccination [7].

Cost-benefit analyses and overall predicted impact on morbidity and mortality might also influence the ethical justifiability of preventive interventions. With the ability to identify a genetic predisposition for adverse events following vaccination, immunization programs might decide to screen for this genetic risk factor. For example, a recent discovery points to a gene variant associated with a significantly increased risk of febrile seizure following vaccination for measles, mumps and rubella (the MMR vaccine) [90]. Febrile seizures are rare and usually benign, raising questions about whether children should be routinely screened for such markers prior to vaccination. If so, and parents are informed of the results, they might decline to vaccinate children who are at increased risk of adverse side effects, risking infection for their children and undermining herd immunity for others. In light of the tremendous public funding and strong support for vaccines from state and federal authorities, it is not clear whether immunization programs have a moral obligation to screen for genetic risk factors, even if screening is not cost-effective.

### Privacy, autonomy and choice

In the United States, clinical decision making has long been tailored to the characteristics, needs and wishes of the individual patient. Along with a physician's obligation to base treatment decisions first and foremost on the wellbeing of the patient come additional responsibilities to respect the patient’s autonomy and privacy. In the context of infectious disease management, individual rights and liberties such as autonomous decision making, freedom of choice and action, privacy, and the right to know or not to know information about oneself can come into conflict with public health priorities. Whereas public health programs may already target people or subgroups with particular risk factors, the possibility of ascertaining (or requiring reporting of) otherwise unobservable genetic risk factors may complicate issues of protection of personal information, privacy and autonomy.

Considerations of privacy and autonomy are being challenged on a massive scale by WGS and whole-exome sequencing (WES), technologies that are expected to contribute to our understanding of host genomics in the context of infectious disease. The planned, as well as unforeseen, uses of the genomic data generated by WGS and WES about individuals and populations raise a range of ethical issues both for initial sequencing and for subsequent use of the data [20–22]. The growing literature on the ethical implications of WGS and WES has so far focused on privacy concerns, data sharing [23], return of results, the management of incidental findings [24] and best practices for obtaining informed consent, at least in the context of research [25,26]. The development and implementation of informed consent policies and practices for the public health uses of WGS information will need to consider (1) whether the information that people ought to have in the context of infectious disease prevention, control and management is different in ethically relevant ways from what is provided in the context of other diseases and behavioral traits, and (2) whether the processes for disclosing information about host genomics should vary, for example, in different parts of the world.

We cannot predict how genetic information might be used in the context of public health or policy decisions; indeed, establishing thresholds for utility in the public health context is made difficult by the probabilistic nature of genomic information. However, we believe it is important to consider ways in which individual genotyping could be used (or mandated) and how its use could affect personal liberties. Genomic data about individuals (their genomic ‘fingerprint’) might be consulted when decisions about prevention and treatment are considered; for example, which vaccine formulation is appropriate, which drugs are likely to be most effective, and what dosage over what period of time. Genomic data about individuals and groups might be consulted during disease outbreaks, in planning for public health programs, or in developing new or assessing existing public health policies; for instance, where are the hotspots for infection (and are these associated with specific pathogen or host genomics), where should vaccines be deployed most urgently, which therapies should be offered to which genomic populations, and where should treatment programs, isolation policies or public health control programs be implemented to halt the spread of infections? Genetic markers of infectivity or likelihood of being a super-spreader could be used to justify quarantine and isolation policies, with the concomitant implications for individual liberty. The value placed on individual autonomy varies in different cultures, so the primacy that it receives in the context of public health planning and decision making, and the role of informed consent, might differ between countries [91,92].

### Social and behavioral impact of genomic information

A number of infectious diseases are transmitted through behaviors that are stigmatizing. Viruses such as HBV, HCV and HIV are commonly transmitted through injection drug use and high-risk sexual practices. Genomic information that can predict the risk of susceptibility to, or transmission of, disease might influence the actual behaviors of individuals in these at-risk groups. For example, the knowledge that a particular genotype decreases the risk for developing chronic hepatitis C might lead to an increase in risk-taking behavior. An overestimate of the predictive value of genetic information emanates from genetic essentialism, the belief that genes are wholly predictive of diseases, behaviors, or traits [93]. The assumption that outcomes are more attributable to genes than is accurate underestimates the importance of individual behavior and contributes to a false sense of security.

In addition to influencing the actual behavior of high-risk individuals, genetic information could affect attitudes and beliefs about the individuals who engage in risky behavior. The knowledge that a genetic variant increases the risk of spreading a sexually transmitted disease might lead to negative judgments about, and marginalization of, individuals who carry that variant. Discrimination against entire subgroups could also occur if, for example, genetic variants were found to correlate with a more favorable vaccine or treatment response, but only in certain ethnic groups; also, drug development might focus on these ‘more responsive’ subgroups.

### Allocation of scarce resources

Disparities in access to critical resources, including preventive or therapeutic drugs, can be due to financial, educational, sociocultural, geographical or environmental barriers. When circumstances, such as a pandemic, create a demand for resources that is greater than the supply, decisions must be made about how to distribute the resources. In the face of shortage, or differential access, genetic information could be used to make triage decisions or decide who receives a vaccine or therapy.

Biomedical research funding decisions could be influenced by the availability of specific genetic information. Special vaccine formulations might be developed and produced for at-risk genetic (‘orphan’) subgroups. It remains to be seen what the implications would be for health insurance coverage and public financing of treatments if vaccines or treatments vary by genotype. The extent to which infectious disease genomics will be translated into benefits for individuals or public health is dependent largely on the allocation of resources for research and development efforts. The majority of research investment comes from high-income countries, whereas the highest burden of infectious disease is in the developing world. The kind of research likely to have the greatest global benefits might not be given funding priority by countries with the greatest resources. Differences in regional investments in genomic science and technology will have important implications for the equitable distribution of benefits and public health impact [94].

### Legal and policy considerations

The legal and policy paradigm in genomics - which places a high value on privacy - can conflict with the public health framework, in which individual rights can be overridden for the benefit of others [95]. All US states have enacted genetic privacy legislation, but the scope of the protections afforded by these laws varies from state to state. The extent to which genetic privacy provisions in these statutes may conflict with state public health laws is unclear. The Model State Emergency Health Powers Act enumerates the powers that will be granted to state and local officials to protect public safety in the event of a public health emergency, and includes provisions related to mandatory vaccination and quarantine [96]. Many states have adopted at least some of the provisions of the model legislation [97].

Host genomic factors could be important in determining: (1) which individuals should be vaccinated in the case of a public health emergency - those who are at highest risk for severe disease; (2) which individuals should not be vaccinated - those who are at high risk of adverse events following vaccination; or (3) which individuals should be quarantined, because of increased risk to themselves or to others. It is unclear whether state emergency powers would override genetic privacy protections in these circumstances, and it is possible that, under current laws, genetic privacy provisions would prevail in circumstances in which a disease outbreak does not rise to the level of a public health emergency.

Similarly, the US federal Genetic Information Non-discrimination Act of 2008 (GINA) [98] forbids discrimination on the basis of genetic information in any aspect of employment, including job placement. Some individuals may be better suited than others to work in high-risk job placements during an infectious disease outbreak because they are more likely to have an adequate response to a vaccine, or because their genotype is associated with a lower risk of developing severe infection. Alternatively, some individuals might have a variant associated with increased risk of severe infection. In both situations, the provisions of GINA may limit the ability to use genetic information to determine which employees would be most appropriate for high-risk job placements in case of an infectious disease outbreak.

Host genomic factors may have additional legal and policy implications. For example, providers may face increased liability for vaccine-related injury in patients whose genotype is associated with greater risk of adverse events following vaccination. Alternatively, those who are found to be at increased risk for adverse events might be exempted from mandatory vaccine laws, potentially affecting herd immunity.

As our knowledge of the role of pathogen and host genomic factors in the prevention and treatment of infectious disease expands, it is critical that we evaluate current legal frameworks to determine the extent to which current genetic privacy laws - for example, both state and federal in the US - may hinder our ability to use genetic information to protect the health of both individuals and the general public. Privacy laws are likely to vary in different countries, and international frameworks for protecting privacy in the context of genomics and infectious diseases will also need to be evaluated.

### Illustrative ELSI issues in genomic applications for particular infectious diseases

#### Ebola

The potential severity of Ebola virus disease, coupled with the absence of effective prevention or treatment, generates interest in determining whether there are host factors that protect people from, or increase their susceptibility to, contracting or spreading the infection. At present, the science of host genomics and pathogen-host interaction is not well understood and, arguably, is not as important as developing treatments. However, imagine if we could identify genetic variants that are associated with an increased likelihood of contracting Ebola, spreading it, having more severe disease or responding to treatment. An ethical tension would arise if we were to consider screening at-risk populations for such variants and using the genomic information to influence a range of clinical and public health decisions. For example, in the absence of effective interventions and sufficient facilities to treat everyone, genetic information might be used to triage patients at greatest risk of severe disease to receive care first. Or we might impose travel restrictions or quarantine only on those who are at greater risk of contracting or spreading the virus. These and other ethical, legal and social challenges need to be considered when designing and conducting genomic research on host factors and host-vector-pathogen interactions in Ebola virus disease.

#### Influenza

Since the transmission of influenza virus does not require close contact, influenza is easier to contract than Ebola and HBV. In the case of pandemic influenza, the severity of the disease and the efficacy of vaccines and treatments are variable, suggesting that limiting exposure is a more promising strategy than relying on interventions. Markers associated with increased susceptibility to infection, severity of disease and response to vaccine could be used to influence workforce decisions. For example, greater responsibilities might be assigned to healthcare workers with genotypes that predict higher resistance to influenza, a greater chance of mild infection, or positive response to the vaccine. Genotyping of healthcare workers might be used, or even required, to determine who can be, or possibly who must be, first responders and, by contrast, who must stay at home. Children might also be screened so as to exclude super-spreaders from going to school. In both of these cases, a higher value would be placed on reducing risk to patients or classmates than on the privacy and autonomy of employees or students, respectively.

#### Hepatitis B

Unlike Ebola, HBV is both treatable and preventable. Although the preventive vaccine is highly effective, 5% of individuals do not respond, and genetic predictors of vaccine non-response are being identified [99]. Screening for such genetic markers could exempt non-responders from vaccination that would otherwise be mandatory, for instance among healthcare workers. Such screening could also influence decisions about access to therapy, especially in settings with limited resources. Treatment for hepatitis B, although very effective, is not curative. If an immunotherapy-based cure is found, treatment might be provided preferentially to individuals with genotypes associated with more rapid disease progression if resources for such therapies are scarce. Also, individuals with genotypes associated with better response to immunotherapy may receive priority for treatment. Alternatively, those most likely to die from these infections might be given priority if vaccines are scarce.

#### Tuberculosis

Like influenza, tuberculosis is airborne and can be transmitted through casual contact. Unlike the diseases described above, tuberculosis can be latent. Although people with latent tuberculosis cannot spread the disease, the ability to test for increased genetic susceptibility to development of active disease could lead to unfair treatment of specific subpopulations that are already marginalized, and could allow familiar ethical issues surrounding the justifiability of ethnic stratification to surface [100–103]. The prevalence of tuberculosis is highest among those in resource-limited settings and crowded environments such as prisons and inner cities. These subgroups could be targeted to undergo testing for susceptibility to active disease, and then treated differentially based on their genotype.

In addition, tuberculosis provides one example in which pathogen and host genomics can be used in combination to identify those at increased risk and to implement measures to control the spread of disease. Sequencing the pathogen can identify the individual who is the source of an outbreak. Those infected by that individual could be pressured to undergo genetic testing and to agree to regular follow-up if they are at increased risk of active infection.

## Conclusions and future directions

We anticipate that genomic discoveries will improve our understanding of infectious disease and inform new strategies for management. Future research directions will shed light on the additional importance of host-pathogen-vector interactions and environmental influences. For example, research on the microbiome - the collective genomes of the microorganisms that inhabit our bodies - is yielding increasing evidence for its role in infectious disease [104,105].

Drawing on four paradigmatic infectious diseases, we have attempted to sketch a view of what the future may hold in terms of ELSI considerations at the intersection of genomics and infectious disease. Some important challenges relate to balancing health-related benefits and harms between individuals and the larger community, minimizing threats to individual liberties, and promoting justice in the distribution of scarce resources and the treatment of marginalized subgroups. While it is too early to identify all of the potential ELSI issues that may emerge in this field, such considerations should be factored into the development of policy recommendations for public health and clinical practice in infectious disease, both domestically and internationally [106]. Attention to ELSI issues could also guide research questions and decisions about public funding of science. This would contribute to the ongoing systematic effort to provide an evidence base for the utility and priority of genomic applications in public health [107,108].

A number of frameworks have been developed for identifying and responding to important issues in the prevention and control of infectious disease [109–111]. An important next step is to develop a framework for spotting and elucidating ELSI issues pertinent to infectious disease genomics that considers the disease characteristics discussed above. We do not have the luxury of waiting until the science matures to consider the potential consequences of these advances. Instead, we must work to predict ELSI issues and be ready to address them as they arise in order to ensure just and sustainable solutions that minimize harm and maximize benefits [112].

## Abbreviations

ELSIs: ethical, legal and social implications
GINA: Genetic Information Non-Discrimination Act
HBV: hepatitis B virus
HCV: hepatitis C virus
MRSA: methicillin-resistant *Staphylococcus aureus*
WES: whole-exome sequencing
WGS: whole-genome sequencing
